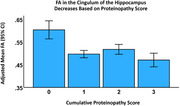# Post‐Mortem Neurodegenerative Proteinopathies are associated with Ante‐Mortem Diffusion Metrics in Medial Temporal Lobe White Matter Tracts

**DOI:** 10.1002/alz70856_100505

**Published:** 2025-12-24

**Authors:** Christopher E. Bauer, Colleen Pappas, Brian T Gold

**Affiliations:** ^1^ University of Kentucky, Lexington, KY, USA; ^2^ Sanders‐Brown Center on Aging, Lexington, KY, USA

## Abstract

**Background:**

Neurodegenerative proteinopathies are strongly associated with volumetric reductions in the medial temporal lobe (MTL). However, significantly less is known about how MTL white matter tracts are affected. Here we explored whether neuropathologically‐confirmed neurodegenerative proteinopathies were associated with antemortem MRI‐derived white matter diffusion properties in MTL tracts.

**Method:**

Post‐mortem brain tissue was collected from fifty‐one older adults (ages 65‐111) through the National Alzheimer's Coordinating Center (NACC) consortium. Alzheimer's disease (AD), TDP‐43, and Lewy Bodies (LB) neuropathology was scored according to NACC criteria. Each participant was scored as having high (>1 ADNC ABC score) or low AD pathology, high (TDP‐43 positive in brain) or low TDP‐43 pathology, and high (LB positive in brain) or low LB pathology. A cumulative proteinopathy score was then calculated based upon the number of pathologies in which each participant was rated to be high (0‐3). All participants also had at least one ante‐mortem MRI scan within 10 years of death. Hippocampal, amygdala, and intracranial volume (ICV) were calculated through FreeSurfer from a T1‐weighted scan. Fractional anisotropy (FA) values were calculated for major tracts of the MTL (cingulum of the hippocampus, uncinate fasciculus, inferior fronto‐occipital fasciculus (IFOF), fornix) through FSL from a diffusion‐weighted scan. All MRI data was harmonized across sites using neuroCombat. Multivariate linear regression models tested whether cumulative proteinopathy scores were associated with antemortem FA in MTL white matter tracts controlling for ADC ID, age at death, time between MRI and death, sex, education, CDR sum of boxes score, and ICV.

**Results:**

There was a significant main effect of cumulative proteinopathy score on volume of MTL structures. Additionally, there was a significant main effect of cumulative proteinopathy score on FA values in MTL white matter tracts. A post‐hoc analysis revealed that this effect was only statistically significant for the cingulum of the hippocampus (*p* <0.001). This effect remained when the volume of the hippocampus and the volume of the amygdala were added as covariates.

**Conclusion:**

Our results suggest that aggregating proteinopathies are linked to alterations in both MTL volumes and tracts. Future work is needed to determine whether changes in diffusion metrics precede changes in volume.